# Twentieth anniversary of the European Union health mandate: taking stock of perceived achievements, failures and missed opportunities – a qualitative study

**DOI:** 10.1186/1471-2458-13-1074

**Published:** 2013-11-14

**Authors:** Nicole Rosenkötter, Timo Clemens, Kristine Sørensen, Helmut Brand

**Affiliations:** 1 Department of International Health, CAPHRI, Faculty of Health, Medicine and Life Sciences, Maastricht University, Duboisdomein 30, Maastricht 6229 GT, The Netherlands

**Keywords:** European Union, EU health mandate, Health policy, Assessment, Qualitative research

## Abstract

**Background:**

The European Union (EU) health mandate was initially defined in the Maastricht Treaty in 1992. The twentieth anniversary of the Treaty offers a unique opportunity to take stock of EU health actions by giving an overview of influential public health related EU-level policy outputs and a summary of policy outputs or actions perceived as an achievement, a failure or a missed opportunity.

**Methods:**

Semi-structured expert interviews (N = 20) were conducted focusing on EU-level actions that were relevant for health. Respondents were asked to name EU policies or actions that they perceived as an achievement, a failure or a missed opportunity. A directed content analysis approach was used to identify expert perceptions on achievements, failures and missed opportunities in the interviews. Additionally, a nominal group technique was applied to identify influential and public health relevant EU-level policy outputs.

**Results:**

The ranking of influential policy outputs resulted in top positions of adjudications and legislations, agencies, European Commission (EC) programmes and strategies, official networks, cooperative structures and exchange efforts, the work on health determinants and uptake of scientific knowledge. The assessment of EU health policies as being an achievement, a failure or a missed opportunity was often characterized by diverging respondent views. Recurring topics that emerged were the Directorate General for Health and Consumers (DG SANCO), EU agencies, life style factors, internal market provisions as well as the EU Directive on patients’ rights in cross-border healthcare. Among these recurring topics, expert perceptions on the establishment of DG SANCO, EU public health agencies, and successes in tobacco control were dominated by aspects of achievements. The implementation status of the Health in All Policy approach was perceived as a missed opportunity.

**Conclusions:**

When comparing the emerging themes from the interviews conducted with the responsibilities defined in the EU health mandate, one can identify that these responsibilities were only partly fulfilled or acknowledged by the respondents. In general, the EU is a recognized public health player in Europe which over the past two decades, has begun to develop competencies in supporting, coordinating and supplementing member state health actions. However, the assurance of health protection in other European policies seems to require further development.

## Background

The Maastricht Treaty from 1992 marked the beginning of the health mandate of the European Union (EU) as enshrined today in Article 168 of the Lisbon Treaty (TFEU, Treaty on the Functioning of the European Union) [1]. The original EU health mandate focused primarily on stimulating cooperation between member states and supporting national actions (Art. 129 (1), Treaty of the European Union (TEU)) [2]. It embodied the Union with only limited legislative powers on health matters. Although this initial mandate was enhanced through subsequent Treaties, today Article 168, still gives the EU relatively circumscribed power in areas of public health (Art. 168 (4), TFEU). Healthcare continues to remain a national competence and in this regard, the EU *“shall respect the responsibilities of the member states for the definition of their health policy and for the organization and delivery of their health services”* (Art. 168 (7), TFEU). Despite the restricted Treaty-based mandate for health, the EU has a relevant role to play in national public health and health systems policies and has expanded its remit in areas beyond the Treaty [3]. Areas affected by EU provisions are extensively described in the literature [4-11]. To illustrate the main developments in the area of what can be called “EU health policy” a timeline is illustrated in Table 1. However, because of its limited legal mandate, some EU legal initiatives were highly contested [12,13].

**Table 1:** Timeline of main developments in EU health policy

	
**Year**	**EU health policy developments before the introduction of a legal EU health mandate**
**1957**	Treaty of Rome: health is not a priority. Two aspects are considered: social security of cross-border workers and occupational health.
**1971**	Regulation (EEC) 1408/71 on the application of social security schemes to employed persons and their families moving within the community (accompanied by implementing Regulation (EEC) No 574/72).
**1985**	EC launches the action programme “Europe against Cancer”.
**1993**	Maastricht treaty: the legal basis for undertaking actions in the field of public health is defined in Article 3(o) and Article 129.
**Year**	**EU health policy developments after the introduction of a legal EU health mandate by the Maastricht Treaty**
**1995**	The European Agency for the Evaluation of Medicinal Products (EMEA), now European Medicines Agency (EMA), has been formed in London.
**1997**	Treaty of Amsterdam: Health Impact Assessment is implemented.
**1999**	Directorate General for Health and Consumers (DG SANCO) is established.
**2000**	Lisbon Agenda recognizes health protection as a prerequisite for economic growth measured with the indicator Healthy Life Years.
**2002**	The European Food Safety Authority (EFSA) has been established in Parma.
**2002**	First Programme of Community action in the field of public health (2003-2008) launched.
**2003**	The Tobacco Advertising Directive 2003/ 33/EC is adopted after the first version has been annulled by the European Court of Justice.
**2004**	Commission decision to set up an Executive Agency for the public health programme. It has the task to manage community action in the field of public health.
**2005**	The European Centre for Disease Prevention and Control (ECDC) in Stockholm is operational.
**2007**	White paper: Together for health: a strategic approach for the EU 2008-2013.
**2007**	Decision for a second Programme of Community action in the field of health (2008-13).
**2011**	Directive 2011/24/EU on the application of patients’ rights in cross-border healthcare has been adopted.

Therefore, one can pose the question of what has been achieved over the last twenty years. It may be argued that, despite its narrow legislative scope, the health mandate has triggered important European actions in certain public health areas like tobacco control [14,15], infectious disease control [16,17], European guidelines [18-20] and the development of an EU public health infrastructure [21]. In recent years, the EC has summarized in annual reports a diverse nature of key public health achievements such as communications and recommendations, health policies, EC co-financed actions and established networks (e.g. high level groups, scientific committees, platforms) [22-24]. However, stakeholders in the field provide examples indicating that public health relevant EU policies such as single EU policy assessments on the Common Agriculture Policy (CAP) [25,26], pharmaceuticals [27], or the Health in All Policy (HiAP) approach [28] do not always meet the expectations of the public health community. In these papers the authors express concerns about potentially detrimental health effects [25,26] or disappointment about the support of the policies and approaches aimed at improving health in Europe [27,28]. Also, EU agencies such as the European Centre for Disease Prevention and Control (ECDC) are described as agencies with a limited legal mandate, competences and resources for EU public health but, at the same time, with promising prospects to develop as a renowned international player in the field [29].

In addition, an evaluation of the EU Health Strategy acknowledges its status as a guiding framework for EC health policies and joint EC and member state actions on health but also identifies the missing impact of the Strategy on other EC policies as well as on member state health policies and actions [30]. Evaluations of the EU public health programmes, which are one of the EC’s financial instruments to implement its strategic health goals, criticize missing prioritization of topics, barriers for participation in projects for some member states and ineffective dissemination of project results [31,32]. Hence, the available evidence of the impact of EU health policies, infrastructure, and actions is elusive, and the identification of the value of public health relevant EU-level actions across all policies is lacking.

In this paper, we aim to explore and provide an overview of influential public health relevant EU-level policy outputs and a summary of policy outputs or actions perceived as an achievement, a failure or a missed opportunity by interviewing key experts in the field. By this, we intend to establish a qualitative indication of which EU health policies have contributed to the improvement of population health in Europe.

## Methods

The study focused on the evolvement of the health mandate since 1992, the year the Maastricht Treaty was signed. The study was carried out in two consecutive phases: (1) qualitative interviews, suitable to identify expert perceptions, and (2) voting on influential and public health relevant EU policy outputs and actions based on nominal group technique. The study adhered to the RATS guidelines on qualitative research [33].

### Study participants

Experts were purposely selected to ensure heterogeneity of opinions. The selection was based on their individual profile and professional affiliation. We selected experts that were renowned in the field due to their current or former affiliation to specific EU-level bodies and institutions, research institutes with EU focus, or EU-level non- governmental organization. Selected experts were actively involved in public health research, policy-making, policy advice or advocacy performed at EU level, internally or externally. In addition, snowball sampling was applied until data saturation was reached. Data saturation was assumed as soon as no new EU public health policy actions and their perceptions were mentioned during the interviews.

The potential participants were contacted between December 2011 and March 2012 by a short information email to identify whether they were interested to participate in the interview study. Of 22 contacted experts, twenty participated in this study, one participant could not confirm participation due to time constraints and another did not respond to the invitation. Of those twenty experts nine belonged to the initial purposively selected sample and eleven were identified during the snowball sampling procedure based on recommendations of already interviewed experts. The majority of experts was affiliated to an institution located in Brussels (n = 10). The composition of the study sample in terms of represented professional affiliations is outlined in Table 2.

**Table 2:** Professional functions of interviewed participants

***Function***	***Number of participants***
EU/national civil servant	7
EU/national politician	2
Academia	5
Public health advisor/advocate	6
**Total**	**20**

Upon agreement by the participant, an appointment for the interview was made and participants received an informative letter with more in-depth information about the goal of the study and an informed consent form in which the voluntary basis of the participation has been clarified and anonymized data handling was assured.

### Qualitative interviews

Interviews were conducted either face-to-face (n = 4) or via telephone, or Voice over IP (n = 16) in the period between January and March 2012 and were held in English, Dutch or German by one of the three principal investigators (NR, TC, KS). The interviews lasted from 35 to 90 minutes. All interviews were audio-recorded, transcribed verbatim and anonymized.

The interviews were performed using a specifically designed semi-structured interview guide. The guide was developed on the basis of previous desktop research and an internal brainstorming session of an advisory research group consisting of the three principal investigators, four senior researchers, and one junior researcher of the Department of International Health at Maastricht University to identify items relevant for investigating expert perceptions on European public health policy. During the course of this process, a list of public health relevant EU policy outputs, processes or procedures that were regarded as achievements, failures or missed opportunities were first gathered individually and then, following a group discussion, a common list was compiled. This output was used to construct the interview guide containing six guiding themes from which open-ended questions were formulated. The guiding themes included (1) a description of the individual role of the expert in European public health, (2) the individual definition of European public health, (3) the assessment of public health relevant EU-level actions as being an achievement, missed opportunity or failure, (4) the formulation of five influential European policy outputs, (5) consequences of European health policy, and (6) the policy process at the European level. The semi-structured interview guide was used as a framework for the interviews and allowed the interviewers to address other relevant topics that emerged during the interviews.

### Analysis

After completion of interviews the three principal investigators initially performed an internal analysis of each separate interview and an analysis across interviews to identify the scope of EU-level actions and experts’ perceptions of these actions as achievements, failures or missed opportunities [34]. Afterwards, a directed (or deductive) content analysis approach [35,36] was applied whereby the initially predefined coding scheme with the main categories of interest (achievement, failure, and missed opportunity) was used to summarize the respective topics and the reasoning that appeared during the interviews. Topics that did not fit into one of these main categories were added as new codes and were organized into new categories. The analysis was jointly performed by the principal investigators using NVivo 9 (QSR International Pty Ltd. Version 9).

Furthermore, the results of the content analysis on achievements, failures and missed opportunities were grouped according to the major common themes in a table to provide an overview of the perceptions of the key informants. Prominent EU-level outputs or actions, which were discussed by almost all respondents, are described in more detail in the results section. Where applicable, we used original quotes to illustrate the views and tendencies of experts’ assessments. The professional profile and study ID of respondents are indicated behind the respective quotes. Quotes which were originally given in Dutch or German were translated into English.

### Nominal group technique

A slightly adapted nominal group technique [37] was used for triangulation purposes. During the interviews, the participants were asked for five influential policy outputs of European health policy-making. Following the finalization of all interviews, all participant nominations were compiled in one list and reoccurring topics were removed. To ensure comparability of policy outputs and actions, we grouped the nominations into categories under the following headlines: (a) secondary legislation and court decisions; (b) soft laws, strategies, and programmes; (c) agencies, centres, organizations; (d) networks, policy platforms, cooperation; and (e) others. Participants were asked in an online survey to select three outputs per category which were, according to their opinion, most influential. Based on the participants’ nominations, a ranking in terms of a frequency distribution of selected influential policy outputs for each category was determined. The online survey was completed by 18 out of 20 participants who took part in the interviews.

### Ensuring quality

The design and analysis of the study was guided by applying Guba and Lincoln’s test for trustworthiness [38]. Credibility and dependability have been ensured by enlarging the sample until saturation was reached in terms of the identification of EU policy actions and their perceptions. Moreover, three researchers in the primary research group in combination with an internal advisory research group were involved with the aim of reflecting upon the study design and critically questioning the findings. Additionally, the primary research group met regularly during the interview period to exchange initial findings and experiences on the interview process. The members of the internal advisory research group were experienced in EU public health policy research or qualitative research methodologies. The confirmability was strengthened by the use of several investigators both in the data collection process and in the analysis phase, combined with the use of triangulation, where interview participants were also asked to participate in the ranking exercise.

### Ethical considerations

The Medical Ethical Committee of the University Hospital Maastricht and University Maastricht declared that no ethical approval was required for this type of research. All participants were informed about their role and rights as study participant prior to their interview participation. All participants provided written or audio-recorded informed consent to be interviewed.

## Results

Overall, respondents consistently mentioned that, during the twenty-year history of the EU health mandate, specific initiators induced change in European public health policy. The most important identified initiators included the Maastricht Treaty with its later amendments, the health-related rulings of the European Court of Justice, and the health crises such as Boviene spongiforme encefalopathie (BSE) and Severe Acute Respiratory Syndrome (SARS). In addition, the internal market provisions with the foreseen free movement of goods, people, services and capital, initiated change with both negative and positive public health impact. Additionally, a set of conditions was identified in the interviews that described and advanced the role of EU health policy as a reference point for public health. These conditions under which EU health policy made progress during the past twenty years were (i) the regulatory power at the EU-level, (ii) EU-led facilitation of cooperation and comparisons across member states; along with (iii) increased capacity building on EU issues and on EU-level (e.g. professionalization, development of interest groups, associations).

### Influential EU health policy outputs

The ranking of influential policy outputs of EU-level health policy-making is provided in Table 3.

**Table 3:** Ranking of influential policy outputs of EU-level public health policy, March/April 2012

**Categories**	**N**
**Secondary legislation and court decisions**	
**Patients' rights decisions by the European court of justice**	**12**
**Directive on patients’ rights in cross-border care**	**10**
**Directive on tobacco advertising**	**9**
Directive on blood safety	5
WHO Framework Convention on Tobacco Control	4
Directive on professional qualifications	3
Regulation 1408/71	3
Directives on tobacco products	2
REACH regulation of chemical substances	2
**Soft laws, strategies and programmes**	
**First and second public health programme**	**9**
**Together for health: 2008-2013**	**6**
**Framework for action in the field of public health**	**5**
**Towards modern, responsive and sustainable health systems (2011/C 202/04)**	**5**
**Europe 2020, Europe’s growth strategy**	**5**
Health in All Policies	4
Information to Patients	3
Open Method of Coordination	3
Solidarity in health: reducing health inequalities	3
Council recommendation on patient safety	**2**
Framework Programmes on health research	2
White paper on governance	2
Green paper on the European workforce for health	0
**Agencies, centres, organizations**	
**European Medicine Agency**	**15**
**European Centre for Disease Prevention and Control**	**13**
**European Food Safety Authority**	**9**
**European Observatory on Health Systems and Policies**	**6**
European Agency for Accreditation of Education in Public Health	1
European Chemicals Agency	1
European Foundation for the Improvement of Living and Working Conditions	1
European Monitoring Centre for Drugs and Drug addiction	1
Social Protection Committee	1
European Commission Food and Veterinary Office	0
European Commission Joint Research Centre institutes	0
Health Intergroup at the Committee of the Regions	0
**Networks, policy platforms, cooperation**	
**European presidencies**	**13**
**Cooperation EC with OECD and WHO**	**8**
**European Health Policy Forum**	**6**
**Network epidemiological surveillance and control of infectious diseases**	**6**
**Network on Health Technology Assessment**	**6**
European Platform for Action on Diet, Physical Activity and Health	3
eHealth Network	2
European involvement in GATS discussion	2
European Committee of Experts on Rare Diseases	2
European Platform against Poverty and Social Exclusion	1
European Innovation Platform on Active and Healthy Ageing	0
European Network of Health Promoting Schools	0
Joint Action Health Workforce Planning	0
**Others**	
**Work on health determinants**	**10**
**Best practice exchange**	**8**
**Scientific reports that influenced European policy-making**	**7**
Budget of the European Union	5
Common Agriculture Policy reform	4
Alcohol regulation	3
HEIDI data tool	2
Initiatives on Roma and excluded groups	1
Project database on DG SANCO website	1
Work on public health core competencies by ASPHER	1

ASPHER: Association of Schoold for Public Health in the European Region.

DG SANCO: Directorate General for Health and Consumer Protection.

EC: European Commission.

GATS: General Agreement on Trade in Services.

HEIDI: Health in Europe: Information and Data Interface.

OECD: Organization for Economic Cooperation and Development.

WHO: World Health Organization.

In the category “secondary legislation and court decisions”, the patients’ rights decisions made by the European Court of Justice (n = 12) were chosen by most of the respondents as influential policy output, followed by the Directive on the application of patients’ rights in cross-border healthcare [39] (n = 10) and the Directive on advertising and sponsorship of tobacco products [14] (n = 9). In the category entitled “soft laws, strategies, and programmes”, the first and second EU public health programmes [40,41] (n = 9) were selected most frequently, followed by the 2008–2013 health strategy “Together for Health” [42] (n = 6). The third rank is shared by three policy outputs: the “Framework for action in the field of public health” [43] (n = 5) which is the Commission’s first proposal setting out EU-level public health after the introduction of the health mandate in the Maastricht Treaty, the Council conclusions “Towards modern, responsive and sustainable health systems” [44] (n = 5), and the current over-arching European strategy “Europe 2020” [45] (n = 5). In the third category on “agencies, centres and organizations”, the European Medicines Agency (EMA, n = 15) ranked top, followed by the ECDC (n = 13) and the European Food Safety Authority (EFSA, n = 9). Among “networks, policy platforms and collaborations”, the European presidencies (n = 13) were selected most often by the respondents, followed by the collaboration of the European Commission (EC), the World Health Organization Regional Office for Europe (WHO-EUR) and the Organization for Economic Co-operation and Development (OECD) (n = 8). Moreover, the three entries on the third rank include the EU Health Policy Forum (n = 6), the network on epidemiological surveillance and control of infectious diseases (n = 6), and the network on Health Technology Assessment (n = 6). The fifth category was not topic specific therefore, work on European level health determinants (n = 10), the exchange of best practices (n = 8), and published scientific reports which influenced EU policy-making (n = 7) were ranked on the first three positions.

### Achievements, missed opportunities, failures

At a glance, the label “achievement” was allocated to the public health mandate as it is laid down in the Treaties, the establishment of EU-level agencies dealing with public health topics and successes in smoking prohibition, food safety and infectious disease control. The label “missed opportunity” was allocated to the insufficient degree to which the HiAP approach is implemented and the ways in which health promotional aspects of alcohol and nutrition were handled. The label “failure” was less often assigned with the missing integration or link to social policies appearing in some interviews under this heading as well as the strength of the internal market which annulled national protective alcohol legislations in some member states. In Table 4, we provide the full list of EU-level outputs or actions which, based on the content analysis and the identified thematic categories, were mentioned as achievements, missed opportunities or failures by the key informants. Due to a broad and divergent spectrum of perceptions, topics almost always shared aspects of achievements, missed opportunities or failures. In the following section, we focus on those EU-level outputs or actions which were mentioned by the majority of respondents during the interviews and allowed us to draw a comprehensive picture on the breadth and the diversity of expert perceptions.

**Table 4:** Summary of public health relevant EU-level actions and their perception as achievement, failure or missed opportunity

**Topics**	**Achievement**	**Missed opportunity**	**Failure**
**Treaty**	Inclusion of the health mandate as enshrined in Article 129 of the Treaty of the European Union.	Missing implementation of a connection/share of power between economic and social EU policy.	
**Directorate general for health and consumers (DG SANCO)**	Existence and persistence of DG SANCO.	DG SANCO is not strong enough to push health in other DGs.	DG SANCO set-up: Missing link to social policy.
	Public health programme.	Public health did not become a key aspect of EU policy.	
		Sustainability development strategy: DG SANCO is not playing an active role in the marketing of the strategy.	
**Cooperation**	Cooperation between EU, WHO and OECD.	Missing connection and joint forces between EC and WHO.	
**EC agencies**	Development of agencies: ECDC, EFSA, EMA, EMCDDA.		
**ECDC**	Legislation on infectious disease control.	ECDC mandate should include responsibilities in risk management of infectious diseases.	
	European Programme for Intervention Epidemiology Training (EPIET).	ECDC profile should cover also non-communicable diseases and SDoH.	
**EMA**	Coordination of the approval of efficacy, safety and quality of drugs.	Cost-effectiveness of pharmaceuticals is not taken into account.	Problem of not being able to tackle pharmaceutical pricing.
		Reversal of the approval of already approved drugs not handled on EU-level.	
**EFSA**	Control of health claims of food products.	EFSA mandate should include/be stronger on health promotion aspects of nutrition (e.g. regulation of advertisement of unhealthy food products).	
	Food safety Directive.		
**Health in All Policies (HiAP) approach**	Health mandate assures that health protection should be guaranteed in all EU policies.	HiAP and Health Impact Assessment have never been implemented fully (tick box exercise).	
	Leads to the discussion of health in other sectors.		
**Lifestyle factors**	Common tobacco legislations in Europe (WHO Framework Convention of Tobacco Control; tobacco product-; tobacco advertising Directive).	The tobacco regulations could have been designed stronger (e.g. more harmonized realisation of smoking prohibition on public places).	Tobacco regulation has some aspects of failure since a strict, general ban is not reached.
	Food safety measures and regulations on health claims.	Missing political will to tackle obesity and related life style factors like unhealthy food products.	
**Health Research Programme**	EU health research budget and outcomes of the programme.		Missing integration of the research programme and EU health research outcomes in public health.
**EU budget**	Largest budget proportion shifted in the Multiannual Financial Framework 2007–2013 from agriculture financing to the funding of cohesion and sustainable growth policies.		
	Health research budget.		
	The use of Structural Funds for investments in health (2007–2013).		
**Internal market provisions**		Internal market rules as source for legislation should be more attentive to health concerns.	Internal market provisions cause problems if member state regulation is more protective regarding health threats than EU regulation.
**Patients’ rights directive**	The patients’ rights Directive in general.	Negotiations on patients’ rights Directive failed to include a strong emphasis on the development of common standards.	
	Effect on cross-border cooperation.		
	Gives legal certainty to policy makers.		
**Common Agriculture Policy**	Policy field which starts to recognize health, e.g. in its white paper on the CAP after 2013 (2009/2236(INI)).	Unrecognized potential for health of the CAP by public health sector.	
**Health information**	Health life years as indicator in the Lisbon strategy.		Missing health information system.
			Lack of morbidity data.
**Different public health topics**			
**health inequalities**	EC communication: solidarity in health: reducing health inequalities in the EU.		
**HTA**	Strengthening of the HTA approach in the EU.	Coordinating cross-country level health technology assessments.	
**Rare diseases**	Coordinated management of rare diseases.		
**Tuberculosis**			Existing drug resistance of tuberculosis as indicator for lacking disease management.
**Health of minorities**	Health of minorities (e.g. Roma) as part of the European agenda.		
**Social care**		Social care is hardly seen as EU competence.	
**Environment (and health)**	Environmental standards set by the EU.	Missing follow-up process on the Environment and Health Action Plan (2004–2010).	
**Information to patients**	Blocking of direct to consumer advertising of prescription-only pharmaceuticals.		
**Governmental issues**	White paper on governance (2001) increased transparency.		
	More standardisation of methods (evaluation of indicators, outcomes, policies) and common language.		
	Increased understanding of the public health community about the impact of EU policies on public health.		
**Industry involvement**			Cooperation with industry influences the health research agenda and policy-making.
			Evidence-based policy-making: the interest of the industry is against public health.

ECDC: European Centre for Disease Prevention and Control.

EFSA: European Food Safety Authority.

EMA: European Medicines Agency.

EMCDDA: European Monitoring Centre for Drugs and Drug Addiction.

HTA: Health Technology Assessment.

DG: Directorate General.

SDoH: Social Determinants of Health.

EC: European Commission.

OECD: Organization for Economic Cooperation and Development.

WHO: World Health Organization.

### EU health policy - an overview

An assessment of the general value of EU-level public health actions over the last twenty years resulted in mainly ambivalent judgments. On the one hand, many relevant activities were performed at an EU-level and the existence of a health mandate contributed to an EU social model. On the other hand, its dependence on political will and economic circumstances influenced the development of EU-level public health policy and led to the perception that more should or could have been achieved within and beyond the possibilities of the current health mandate.

### The role of the Directorate General for Health and Consumers

The establishment of the Directorate General for Health and Consumers (DG SANCO) in 1999 as an independent, formal structure for EU health policy was generally discussed as an achievement. The formation of DG SANCO, and thus, the political decision to separate the health dossier from DG V, the former DG with the responsibility for health policy as well as a focus on employment and social policies, was controversially perceived. The establishment of DG SANCO led, on the one hand, to a more mature health policy field.

“…the DG V was a big DG and then DG SANCO became separate from that. Health had its own commissioner, its own opportunity to protect itself and public health benefits.” (#15, public health advisor/advocate)

On the other hand, aspects of failure were mentioned regarding the detachment of health and social policy at EU-level. According to the respondents being separated led to a loss of collaboration for more holistic health policies and actions in health systems and healthcare at EU-level.^a^

Following the formation of DG SANCO, it was seen as a beneficial way forward for the DG to shift its sectorial policy approach from a focus on specific topics such as cancer, drug dependence, health monitoring, accidents and injuries, or pollution-related diseases, to a horizontal one with the formulation of the first health strategy with three cross-cutting objectives: health information, health threats and health determinants [46].

“And this was an important moment in time where the sectorial approach to AIDS, cancer and other issues has been reduced gradually and that more the integral horizontal approach, which was applied at that time already in all member states – hence Europe was running behind in that sense, but ultimately was embraced and taken as guideline for the framing of all sorts of public health actions. “(#02, EU/national civil servant)

Since it fostered more visibility of the public health field and closer cooperation by financing projects, joint actions and research across Europe, the Public Health Programme of DG SANCO was commonly discussed as being supportive to the development of European public health and the mobilization of the public health community.

Aspects of missed opportunity became relevant when assessing the representation of health in other EU policies.

“I don’t know what exactly the reason is, but they [DG SANCO] are not strong enough to push for health in [the other] DGs. The obvious example is the latest EU 2020 strategy, you cannot find reference to health anywhere it’s really a disaster, because of [the] weak DG SANCO. Health is not among the headline targets, it’s not among the flagships.” (#15, public health advisor/advocate)

While the cooperation with other DGs was recurrently discussed as problematic, the potential for the “Partnership on Active and Healthy Ageing” under the European Innovation Union appeared as a unique theme and was regarded as an achievement for strengthening health policy on the general EU policy agenda.

“…it is now coordinated under the Innovation Union [of Europe2020], and so Commissioner Neelie Kroes [DG Connect and Vice-Commissioner] is as much in the lead as Commissioner John Dalli [DG SANCO]. And that’s a good sign, if we can get more of those sorts of partnerships on specific policies, then I think, we’ll get a better understanding.”(#04, EU/national politician)

With regard to assessing the status of DG SANCO cooperation with other international policy actors, respondents had mixed perceptions. Whereas some argued that DG SANCO’s collaboration with international organizations like the WHO-EUR or the OECD is improving and therefore, can be considered as an achievement, others asserted that this collaboration was not sufficiently established and can therefore be categorized as a missed opportunity.

### The role of health agencies: the example of ECDC

The establishment and the work of EU public health agencies like the EMA, the ECDC, the EFSA, and the European Monitoring Centre for Drugs and Drug Addiction (EMCDDA) were regarded as an achievement and as an important step forward towards the strengthening of the European dimension in health. The work and the scope of the agency mandates was a recurring topic and subject to diverging perceptions. As an example, in the case of the ECDC, its development was assessed as an achievement whilst its scope was considered a missed opportunity.

The bioterrorism attacks on the United States of America in 2001 and the SARS crisis led to calls for better international coordination of infectious disease surveillance and the establishment of ECDC in 2004 [47]. Hence, the setting up of ECDC was commonly perceived as an achievement, since it gave preceding EU actions in infectious disease control a formal structure and maintained actions in the field. Also, the close collaboration with the respective national public health agencies during outbreaks and in negotiating and developing common guidelines for infectious disease control were regarded as an important task of the ECDC.

However, a number of respondents were critical of the scope of the ECDC mandate and thus, looked at this as a missed opportunity. Questions were raised on whether the ECDC’s responsibility in surveillance, risk assessment and training are sufficient or if additional responsibilities in risk communication and management were needed to assure full stewardship during and in the prevention of health crises.

“I suppose the flu epidemic […]. That should be put on the table not only as a missed opportunity, big failure, having put ECDC at the center of the development, but the ECDC is not authorized to risk communicational management as you know. So, in that sense, it is a failure that member states were not able to coordinate in this very important public health area and use the EU institution, either ECDC or WHO to do that.” (#03, public health advisor/advocate)

Moreover, interview participants reported tensions between member states and EU agencies regarding the transfer of responsibilities from national to EU-level.

“And the member states are very reluctant to hand over power regarding public health to the Commission, or to Brussels. Now if you focus on infectious diseases, that is much better because they understand that there is a need, but again it is not easy.” (#30, EU/national civil servant)

Since the largest burden of disease in the EU is caused by non-infectious rather than infectious diseases, a call was put forward to further increase the mandate of ECDC to all public health relevant aspects and not focus only on infectious diseases.

### Health in All Policies (HiAP)

The HiAP approach was generally assessed as an achievement regarding its potential to address health determinants outside the health sector.

“[the article on the health mandate] is very important, because thereby a mandate is created that the Commissioner for Health and Consumer Affairs […] approaches his colleagues whenever they make new legislation to ensure that the health protection dimension is guaranteed; it gives partly a mandate to break into the policy and law development in sectors which in principle do not have any links with public health. […] this is very difficult. But its potential is very strong.” (#2, EU/national civil servant)

However, in regard to its degree of implementation participants commonly perceived HiAP as a missed opportunity. Health Impact Assessment, the implementing tool to HiAP, was regarded as a *“tick box exercise” (#03, #15, both public health advisor/advocate)* rather than a thorough consideration of health in other policies areas. Explanations given during the interviews demonstrated that conditions to achieve HiAP seemed not to be established yet and that there seems to be difficulty in bringing DG SANCO interests in line with the interests of other DGs without over-emphasizing the health aspect. Political assertiveness in convincing other Commissioners and DGs about the relevance for intersectoral cooperation was perceived to be lacking, even though an Inter-service group on Public Health with the participation of more than twenty EC departments was established for this purpose.

### Approach to life style factors

Generally, the work regarding tobacco was regarded as an achievement of how European health policy-making effectively addressed a life style risk factor for health.

*“The progress around tobacco [Directive on tobacco advertising, Directive on tobacco products, transparency register], the fact that we have a piece of international law on tobacco [WHO Framework Convention on Tobacco Control] is massive and that was European led*.” *(#05, public health advisor/advocate)*.

This quote echoed the perception of the majority of respondents who emphasized the leading role of the EU regarding the support and commitment to the WHO Framework Convention on Tobacco Control. Moreover, it was argued that the achievements regarding the regulation of tobacco advertising and smoking prohibition in public places would not have been achieved by single member states independently and thus this was a common achievement initiated and supported by European cooperation. Nevertheless, aspects of a missed opportunity or even failure were mentioned in this regard since some would have appreciated stronger legislative measures to achieve a more harmonized realization of smoke-free legislation across the EU.

It was considered that the achievements recognized in EU tobacco legislation were missed in the regulation of other health-related life style factors such as nutrition and alcohol. Whilst regulations in the area of food safety were generally acknowledged as an achievement by preventing food-borne health threats; a potential mandate to address the composition of food and thereby, prevent, *inter alia* obesity or non-communicable diseases seemed to be neglected and was labeled as a missed opportunity.

“…food safety has been majorly put forward over the last twenty years, in the sense that we know that the food will not be contaminated. But then it is a missed opportunity in the sense that beyond food safety there is health promotion and then one wanted the Union to have more powers to regulate issues on the content of saturated fat for instance or the percentages of sugar and so on.” (#03, public health advisor/advocate)

With regard to governmental activity on these issues, the EU Platform for Action on Diet, Physical Activity and Health was named as an example of an achievement as well as a failure.

“I think the diet platform […] can be seen as a failure and opportunity. […] If we had not created that platform then arguably the issue wouldn’t have been tackled at all. And in a way that has been really brought some issues of complexity to the political discussion around issues around marketing of food, around self-regulation, reformulation, some of the initiative like salt in diet has come as a commitment from that platform.”(#05, public health advisor/advocate)

The failure aspect of the EU Platform for Action on Diet, Physical Activity and Health was related to the perception that a platform is a rather weak policy instrument and that more political will to tackle these issues with stronger EU policy or legal instruments would have had more impact.

Additionally, the lack of timely cooperation of public health professionals with other sectors such as agriculture was raised as missed opportunity. It was illustrated that agriculture policy has public health relevant links regarding affordability, accessibility, and the availability of food. However, it was also argued that this cooperation has been developed further over the recent years.

“And it is correct, that the Common Agriculture Policy has not been taken up health in the beginning, but by now they are doing this very consciously. […] Thus, I really see an improvement; I actually do not see a situation anymore in which health was influenced really negatively [by the Common Agriculture Policy].” (#26, EU/national civil servant)

### Internal market provisions

Internal market provisions were perceived as ambivalent by the respondents. The EU is based on internal market rules that also affect EU health policy.

“The engine of European health policies is still the market.”(#23, academia)

However, the influence of EU market regulations, for example on alcohol policies, was perceived as a failure when member states had more protective and stricter national legislation as was the case in the Nordic countries. Respondents claimed that EU internal market regulations that are more attentive to health issues would be appreciated in this case. Moreover, the potential given by Articles 36 and 114 of the TFEU, which put limitations on the Single Market, was mentioned and it was perceived as a missed opportunity that this potential had not been fully taken up by public health experts:

“[…] the public health aspect, which is written into Article 95 [now Article 114, TFEU] on the internal market, you can put limits on the internal market on the grounds of public safety, public morality and public health, is almost never used. What if DG Internal Market was turned into our greatest weapon?” (#32, public health advisor/advocate)

This was positively exemplified by the case of tobacco control which applied internal market rules for public health purposes to assure harmonized labeling, packaging, nicotine content, etc. across the EU. However, the application of the health argument to put limitations on the internal market rules was also perceived as being negatively connoted by non-public health experts:

“If you just go to the DG Internal Market and grab the first person you see and ask them what public health means, they will tell you it’s the exception member states use to defend local weird monopolies on peculiar alcohol, or something like that. It’s an exception to a rule.” (#19, academia)

### Patients’ rights Directive

The recent EU patients’ rights Directive in cross-border healthcare [39] was mainly regarded as an important achievement. This assessment was not necessarily driven by satisfaction with the scope of the Directive but, instead, because it is the first EU secondary legislation ever enacted specifically on healthcare.

“[…] the cross-border Directive will turn out to be incredibly important. Particularly because it is so symbolic important if you like because it does represent really the first time that the EU has got any concrete in relation to healthcare as opposed to public health. The consequences of this remains to be seen.” (#29, EU/national civil servant)

“Therefore, I see the patients’ Directive as a true success from a legislative perspective” (#23, academia)

The achievement aspect was supported by perceptions that the Directive will lead to more cross-border cooperation and will have an impact on quality of care as well as on priority setting in healthcare and the packaging of healthcare services. Thereby, it was expected that the Directive will not only influence people who seek healthcare services in other countries but also those who seek services in their home country. In this regard, some expected that the Directive would also ultimately empower patients as consumers of healthcare services.

“The cross-border Directive […] will have consequences of more consumer empowerment, consumer rights, patient rights, more consumer participation and more literacy,…”(#03, public health advisor/advocate)

However, there were also critical voices that interpreted the Directive, as targeting a limited segment of the European population and hence, potentially increasing health inequalities. These respondents also questioned the willingness of the general population to seek healthcare treatment outside their home country. Furthermore, respondents were critical of the extent to which more EU involvement in healthcare of member states would lead to quality assurance in general:

“It is positive in the way people can be treated where they want, but it is still my point of view that we […] want to have our own level of quality and we don’t want others to decide what level it should be. Perhaps, because we have a very high quality […]. But of course we don’t mind to tell others about it, we don’t mind others to come in, we don’t mind to help others to get the same standard - that is cooperation, so I always say I love cooperation but I do mind the harmonization.”. (#18, EU/national politician)

## Discussion

This study provides an overview of public health relevant EU-level actions of the past twenty years. We outlined the diverse nature of expert perceptions on key developments in the field and provided a ranking of the most influential achievements. The assessment of outputs or actions being an achievement appeared across and within interviews along with assessments of outputs or actions being a missed opportunity and less often a failure. Thereby, it turned out that the EU public health field has significantly developed its organizational structures (DG SANCO, supranational agencies dealing with public health) and incorporated public health topics like infectious disease control and tobacco control, whereas the HiAP approach still included untapped potential. This finding confirms “The Challenge of Implementation” [48] of the HiAP concept in the EU [28,49]. Given the fact that according to Article 9 and Article 168 (1),TFEU [1], a high level of human health protection should be ensured within all EU policies and actions, it was seen as a weakness that the uptake of health consideration in the general EU policy-making process was low [28]. Ollila described the importance of communication and cooperation strategies for a successful realization of the HiAP approach [49]. The deficiency of these strategies was raised during the interviews which indicated that the performance of EU health players is perceived to be particularly poor in this regard.

### Concordance of interview responses with tasks formulated in the health mandate of the EU

Interestingly, the study indicated that the Treaty-based tasks such as support of cooperation between member states, development of guidelines and indicators, best practice exchange, and periodic monitoring and evaluation on EU-level public health to ensure *‘a high level of human health protection’*[1] were only partially perceived as fulfilled or acknowledged by the interviewed experts. Thematic discussions on actions or policies related to the development of guidelines and indicators appeared with regard to infectious disease surveillance and management of rare diseases but were not a major theme across interviews. The EU-level task to promote best practice exchange among member states was regarded as influential, which is represented by a top position in one of the rankings presented in this paper. With regard to the task of establishing monitoring and evaluation structures, some respondents perceived the status of the EU health information system rather as a failure. This corresponds to observations in the literature indicating that although ground work such as the development of a common EU health indicator set is acknowledged [50,51] further efforts are needed to implement and maintain health indicators [51] and to develop a permanent and sustainable EU public health monitoring and reporting infrastructure that supports decision making in public health on EU level [50,51]. Respondents agreed that cooperation in the area of public health between member state representatives and experts as well as with other stakeholder groups has increased and has been facilitated by the EU through various projects, networks, forums, and platforms. This trend was mainly positively perceived since it supported EU-level public health policy by accumulating and exchanging knowledge, generating public support and a legitimacy to act on certain fields [3]. This finding is corroborated by the literature on the potential of new governance instruments for health- and social policy-making at EU-level [11,52-54]. However, these new governance instruments can also be regarded as a rather strategic investment of the EC to keep topics on the agenda until a political window of opportunity opens but as an ineffective policy tool to enforce and implement action in due course [6]. The collaboration of a diverse set of stakeholders as it is the case for example in the EU Platform for Action on Diet, Physical Activity and Health can lead to actions that constitute rather a compromise of various interests. Consequently, the results might be disappointing from the viewpoint of public health experts [6,55]. A final judgment on the impact of facilitating collaboration is to be awaited and may only be made in the long term future. It will require different ways of measuring ‘impact’ compared to the analysis of domestic adaptations when implementing EU hard law [56].

### Characteristics of EU health policy that influenced experts’ perception

The assessments of EC tasks for public health policy making have been influenced by characteristics like the subsidiarity principle throughout several interviews. On the one hand some participants were in favor of more EU influence on health policies and their implementation. In their view integration and harmonization of health policy did not reach far enough and hence their perception of actions was dominated by the category ‘missed opportunity’. On the other hand some experts were in favor of keeping certain health issues like health care as national responsibility which led to a perception of too much EU involvement and a negative perception of the evolvement of the health mandate.

Public health has a cross-cutting nature and cooperation across DG’s often poses difficulties. Therefore, convincing evidence is required to demonstrate the health impact of policies outside the health domain and strong partnerships are needed to counter strong industrial lobbying groups [6,57,58]. The ease of cooperation and the potential to achieve policy coherence between DG SANCO and DGs with stronger regulatory competences like the internal market (e.g. regarding tobacco, pharmaceuticals) or agriculture policy (regarding food safety, subsidies of unhealthy versus healthy food products) represented another characteristic that influenced the individual perception of EU public health policies. Experts who assessed the value of EU health policy actions under the reality of a rather weak health mandate were more likely to perceive EU actions as achievements. This was in contrast to others who strove for more appreciation of social and health matters in EU policies and who perceived a lot of missed opportunities or failures in this regard as the power of the EU was too weak to realize change and to fulfill the objective of the health mandate to ensure human health protection for citizens in the EU.

In summary, underlying themes such as cooperation among European public health professionals, increasing institutionalization, and characteristics such as the issue of subsidiarity or the possibilities to cooperate across EU policy domains influenced experts’ perceptions throughout the topics presented in this paper. These conditions and characteristics are part of what Lamping called the “chaordic dynamics” of European integration in the field of health policies [3]. As our study demonstrated EU health policy does not demonstrate a clear-cut success since the logic of action in the field can involve diverging interests. Nevertheless, the EU public health has quite systematically developed in terms of scope and impact beyond the original mandate.

### Limitations

The ranking of influential policy outputs provided indications on important developments in EU public health policy. However, even though we categorized the outputs, they sometimes differed in character and power which might have led to imbalanced judgments.

Additionally, we received different reasons for labeling EU-level actions or policies as achievements, missed opportunities or failure for public health. Some were identified because they increased the strength or value of EU-level public health policy, whereas, others were identified because they impacted the health of the European population.

The findings of the study may not be empirically generalizable since they were closely linked to qualitative individual perceptions and the settings that participants belonged to. However, we are confident that the broad range of profiles of the experts has ensured the diversity of perceptions on the topics varying from achievement to missed opportunity and failure. Moreover, given that participants were generally active in health policy at EU-level and mainly positive about the EU, this could also have influenced the obtained results to some extent.

## Conclusion

EU public health policy is subject to divergent perceptions of how successful or unsuccessful specific topics have been tackled and how far European integration in public health policy should go. From the findings, it is unequivocal that the EU has strengthened its role over the past twenty years in supporting, coordinating, and supplementing member states’ actions on public health issues as laid down in Article 168 (2), TFEU. The EU is now a recognized player in public health in Europe. However, when it comes *“to the promotion of a high level of […] protection of human health […]in defining and implementing its policies and activities”* (Article 9, TFEU), further work is needed to achieve the full potential of the EU health mandate.

## Endnote

^a^Also several EU member states disconnected on national level the Ministry of Health from Social Affairs. At the time of writing only seven out of 28 EU member states organized health and social affairs within one ministry (Spain, France, Sweden, Finland, Estonia, Greece, the Netherlands).

## Abbreviations

ASPHER: Association of Schools for Public Health in the European Region; BSE: Boviene spongiforme encefalopathie; DG: Directorate General; DG Connect: Directorate General for Communications Networks, Content and Technology; DG SANCO: Directorate General for Health and Consumers; EC: European Commission; ECDC: European Centre for Disease Prevention and Control; EFSA: European Food Safety Authority; EMA: European Medicines Agency; EMCDDA: European Monitoring Centre for Drugs and Drug Addiction; EU: European Union; GATS: General Agreement on Trade in Services; HEIDI: Health in Europe: Information and Data Interface; HIA: Health Impact Assessment; HiAP: Health in All Policies; HTA: Health Technology Assessment; OECD: Organization for Economic Cooperation and Development; SARS: Severe acute respiratory syndrome; SDoH: Social Determinants of Health; TFEU: Treaty on the Functioning of the European Union; WHO-EUR: World Health Organization- Regional Office for Europe.

## Competing interests

The authors declare that they have no competing interests.

## Authors’ contributions

All authors were involved in setting up the study. NR and TC coordinated the study. NR, TC and KS carried out the interviews, performed the analysis, and interpreted the results. NR drafted the manuscript. All authors revised the manuscript and approved the final version.

## Pre-publication history

The pre-publication history for this paper can be accessed here:

http://www.biomedcentral.com/1471-2458/13/1074/prepub
